# Degradability of cross-linked polyurethanes based on synthetic polyhydroxybutyrate and modified with polylactide

**DOI:** 10.1007/s11696-017-0218-4

**Published:** 2017-06-14

**Authors:** Joanna Brzeska, Magda Morawska, Wanda Sikorska, Agnieszka Tercjak, Marek Kowalczuk, Maria Rutkowska

**Affiliations:** 1 Department of Commodity Industrial Science and Chemistry, Gdynia Maritime University, 83 Morska Street, 81-225 Gdynia, Poland; 2 Centre of Polymer and Carbon Materials, Polish Academy of Sciences, 34 Sklodowska-Curie Street, 41-819 Zabrze, Poland; 3 Group ‘Materials+Technologies’ (GMT), Department of Chemical and Environmental Engineering, University of the Basque Country (UPV/EHU), Plaza Europa 1, 20018 Donostia-San Sebastián, Spain; 4 School of Biology, Chemistry and Forensic Science, Faculty of Science and Engineering, University of Wolverhampton, Wolverhampton, WV1 1SB UK

**Keywords:** Degradable polymers, Cross-linked polyurethanes, Synthetic polyhydroxybutyrate, Polymer blends, Polylactide

## Abstract

In many areas of application of conventional non-degradable cross-linked polyurethanes (PUR), there is a need for their degradation under the influence of specific environmental factors. It is practiced by incorporation of sensitive to degradation compounds (usually of natural origin) into the polyurethane structure, or by mixing them with polyurethanes. Cross-linked polyurethanes (with 10 and 30%wt amount of synthetic poly([*R,S*]-3-hydroxybutyrate) (R,S-PHB) in soft segments) and their physical blends with poly([d,l]-lactide) (PDLLA) were investigated and then degraded under hydrolytic (phosphate buffer solution) and oxidative (CoCl_2_/H_2_O_2_) conditions. The rate of degradation was monitored by changes of samples mass, morphology of surface and their thermal properties. Despite the small weight losses of samples, the changes of thermal properties of polymers and topography of their surface indicated that they were susceptible to gradual degradation under oxidative and hydrolytic conditions. Blends of PDLLA and polyurethane with 30 wt% of *R,S*-PHB in soft segments and PUR/PDLLA blends absorbed more water and degraded faster than polyurethane with low amount of *R,S*-PHB.

## Introduction

Last decades have brought a significant increase in the amount and kind of renewable substrates used to obtain polyurethanes (PUR). Use of natural materials is one of the methods of making polyurethane materials more biocompatible and biodegradable. Cross-linked polyurethanes are modified chemically by introducing bio-based substrates, such as: saccharides (Okoli et al. 2014; Zia et al. 2016a), oils (Arniza et al. 2015; Ionescu et al. 2016), bio-based polyesters (Zhang et al. 2015), and others (Silva et al. 2009; Datta and Głowińska 2014) into the PUR structure. Properties of polyurethanes can be also modified by physical blending of linear and cross-linked PUR with compounds existing in nature or obtained by the biosynthesis, like peptides (Zuber et al. 2015), polysaccharides (Saralegi et al. 2014; Zia et al. 2016b; Brzeska et al. 2015a) and polyhydroxyalkanoates (Martínez-Abad et al. 2016).

Introducing bio-compounds into the polyurethane structure allowed naming it as bio-polyurethane, whereas mixing polyurethane with natural material—as bio-composite (or bio-blend) (Aranguren et al. 2015). Degradable polyurethanes (linear and cross-linked) can be also obtained from chemically synthesized degradable substrates, e.g., synthetic poly([*R,S*]-3-hydroxybutyrate) (R,S-PHB) (Brzeska et al. 2015b, 2016). R,S-PHB is synthesized by anionic ring opening polymerization of ß-butyrolactone and it is a promising substrate for degradable materials. Its amorphous character Amorphousness and the presence of its monomers in nature make the *R,S*-PHB susceptible to degradation under natural conditions.

Polylactide-based polymers are commonly used as degradable material for packaging and medical applications, because they are degradable and bioassimilated in a human body and in the natural environment (Vert 2015). Hydrolysis is the main pathway of polylactide (PLA) degradation. Degradation of ester moiety in aqueous medium leads to the formation of carboxyl and hydroxyl hydrophilic end groups. The rate of degradation depends on the polymer character and structure, and thus on the ease of penetration of water into the polymer network. Amorphous, hydrophilic polymer with low molecular weight and density is degraded faster than that which is crystalline, hydrophobic and with high molecular weight and density.

Properties of PLA are strictly connected with its stereoregularity (Nampoothiri et al. 2010). PLA homopolymers (based only on l-lactide or d-lactide) are semi-crystalline polyesters, whereas meso-lactide (d,l-lactide) and racemic-lactide with the amount of _D_-lactide below 93% give amorphous PLA materials (Raquez et al. 2013).

Kinetics of polymer degradation is affected both by the environment conditions and by the polymer structure and their physical properties. Properties and degradability of physical blends of linear polyurethanes and polylactide are quite often investigated (Imre et al. 2013; Brzeska et al. 2015c; Jašo et al. 2015; Yu and Huang 2015; Jing et al. 2015), especially with reference to their potential usability for medical applications (Grzesiak et al. 2015; Saini et al. 2016). Whereas, blends of cross-linked polyurethane with polylactide are tested much less frequently (Gurunathan et al. 2014).

In this paper, the influence of structure, density and swelling of cross-linked polyurethanes (based on synthetic R,S-PHB) on degradability of their blends with poly([d,l]-lactide) (PDLLA) in hydrolytic and oxidative solutions is presented.

## Experimental

### Materials

R,S-PHB was obtained by anionic ring opening polymerization of *ß*-butyrolactone initiated by 3-hydroxybutyric acid sodium salt/18-crown-6 complex at room temperature and terminated with 2-bromoethanol (Arslan et al. 1999). Before the synthesis of polyurethanes, oligomeroles of R,S-PHB (*M*_n_ 1700) and PCL_triol_ (*M*_n_ 900, Aldrich) were dried by heating at 60–65 °C under reduced pressure (1.4 hPa). Other reagents were previously purified: 4,4′-methylene dicyclohexyl diisocyanate (H_12_MDI) (Alfa Aesar) was vacuum distilled, whereas chain extender 1,4-butanediol (1,4-BD) (Aldrich) was distilled azeotropically with benzene. Solvent *N,N*-dimethylformamide (DMF) (Labscan Ltd) was dehydrated over diphosphorous pentoxide (P_2_O_5_) and distilled under low pressure before the synthesis. Catalyst: tin(II) octanoate (Sn(Oct)_2_) (Akra Chem.) was used as received.

**Scheme 1:**
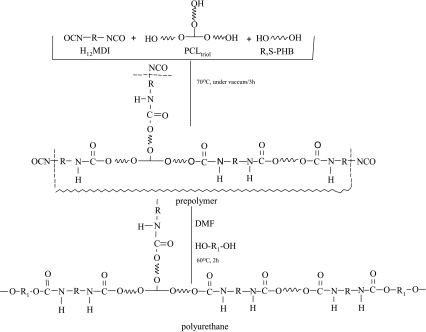
Scheme of PUR preparation

The synthesis of polyurethanes was carried out in a two-step reaction (as showed in Scheme 1), with the molar ratio of NCO:OH = 4:1 in the prepolymer step (Brzeska et al. 2017). Prepolymer of polyurethanes was synthesized for 3 h at 70–75 °C under vacuum with mixed oligomeroles (in the weight ratio of R,S-PHB:PCL_triol_ = 10:90 or 30:70) and H_12_MDI. NCO-terminated prepolymer was dissolved in DMF, and next its molecular weight was increased by reaction with chain extender (1,4-BD) for 2 h at 60 °C. Before pouring the polyurethane solution on Teflon plates it was blended with poly([d,l]-lactide) (*M*_w_ 18,000–28,000, Aldrich) dissolved in DMF. Blend foils were formed by heating at 80–105 °C in the vacuum heater for 6 h.

Polyurethanes (non-blended with PDLLA) were also synthesized and investigated to estimate of PDLLA influence on their properties.

### Methods

#### Differential scanning calorimetry (DSC)

Thermal properties of polyurethanes and their blends were determined using the Setaram thermal analyzer. Indium and lead were used for calibration. The specimens were sealed in aluminum pans and scanned from 20 to 200 °C with the heating rate of 10 °C/min. All experiments were made in a flow of dry N_2_. The melting temperatures (Δ*T*_m_) were determined as a minimum of melting endotherms whereas the melting enthalpies (Δ*H*_m_) were done by integrating the melting peaks on DSC curves.

*Morphology* of polymer surface was investigated by atomic force microscopy (AFM). AFM images were obtained operating in tapping mode (TM–AFM) with a scanning probe microscope (Dimension ICON Bruker) equipped with an integrated silicon tip/cantilever having a resonance frequency ~300 kHz. Taken into account the similarity between height and phase AFM images, only AFM-phase images are shown here. The average roughness (*R*_a_) was calculated using height AFM images. The roughness values for each investigated sample were taken from three independent areas.

#### Density

Density of polyurethanes was estimated using analytical balance equipped with the density determination kit. The measurements were repeated five times for each polymer.

#### Oil and water sorption

Polyurethanes and blends samples were immersed in sunflower oil at 37 °C for 24 h and next they were weighed after wiping from the oil with filter paper (Szelest-Lewandowska et al. 2002). For water sorption estimation, the investigated polymers were immersed in deionized water for 14 days at 37 °C. Next the swollen samples were gently blotted with filter paper and weighed.

#### Swelling

Swelling of polyurethanes and their blends was determined in chloroform (CHCl_3_). Samples (1 cm^2^) were immersed in CHCl_3_ at room temperature. After 24 h, the swollen sample was weighed directly after gently blotted with filter paper.

Oil and water sorption and swelling in chloroform were calculated from the weight after incubation (*w*_*i*_) and the initial weight (*w*_0_) by:

Sorption % = (*w*_*i*_ − *w*_0_)/*w*_0_ × 100%. The results were the average of three measurements.

#### Degradation in buffer solution (PBS)

Hydrolytic degradation of polyurethanes and their blends was carried out for 4, 12, 24 and 36 weeks, using the phosphate buffer solution (PBS, pH = 7.01), containing sodium azide (0.02%) as the bacteriostatic agent. The pH of the aging medium was checked every 2 weeks and the solution was replaced if the pH had changed by more than 0.5 (Glarner and Gogolewski 2007).

#### Degradation in oxidative solution (OX)

Oxidative degradation of polyurethane samples was carried out for 2, 4, 12 and 16 weeks, using the oxidative solution of 20% w/w hydrogen peroxide in the 0.1 M cobalt chloride solution. The solution was changed every week to maintain constant concentration of radicals. One week was selected as an appropriate interval to replace the solution since the half-life of hydrogen peroxide at 37 °C was measured to be about 7 days (Feng and Li 2006).

The progress of degradation of the samples in both environments was monitored by observing changes in their mass, the surface morphology and in thermal properties.

## Results and discussion

Two cross-linked polyurethanes were synthesized as shown in Scheme 1.

Polyurethanes were named according to the percentage of R,S-PHB in the soft segments, which was 10 and 30% by weight for PUR10 and PUR30, respectively.

The nomenclature and composition of polyurethanes and their blends with polylactide are presented in Table 1.

**Table 1:** Name and composition (with wt% of substrates in total mass) of polyurethanes and their blends with polylactide

Sample	Substrates used for soft segments building (wt%)		Substrates used for hard segments building (wt%)		Amount of PDLLA in blend (wt%)
	R,S-PHB	PCL_triol_	H_12_MDI	1,4-BD	
PUR10	3.3	29.3	53.6	13.6	0
PUR10/PDLLA					5
PUR30	10.7	24.8	51.4	13.1	0
PUR30/PDLLA					5

The weight ratio of hard to soft segments in both polyurethanes was similar. They were only differed in amount of R,S-PHB and PCL_triol_ in soft segments.

Density and swelling of polyurethanes and their blends with polylactide in vegetable oil and in chloroform are presented in Table 2.

**Table 2:** Density and swelling in oil ad chloroform (±standard deviation, SD) of polyurethanes and their blends with polylactide

Sample	Density (±SD) [g/cm^2^]	Oil sorption (±SD) [wt%]	Degree of swelling in CHCl_3_ (±SD) [wt%]
PUR10	1.07 ± 0.03*	0.5 ± 0.1*	573 ± 68
PUR10/PDLLA	1.10 ± 0.04	1.0 ± 0.4	348 ± 23
PUR30	1.06 ± 0.03*	0.7 ± 0.1*	842 ± 101
PUR30/PDLLA	1.09 ± 0.01	1.5 ± 0.1	97 ± 13

* Data presented in (Brzeska et al. 2017)

Density of the obtained polyurethanes did not exceed the value 1.07 g/cm^2^ (Table 2). It is known that the presence of cross-links in the polymers structure reduces mobility of macrochains, and restrains their interchain reactions (connected mainly with creation of hydrogen bonds), what decreases the polymer density. Blending polyurethanes with PDLLA increased the mobility of chains and allowed on their better packing which was the reason of density rise. An increase of density after PURs blending with PDLLA simultaneously reduced chloroform swelling (Table 2). Despite of high swelling of samples in chloroform cross-linked polyurethanes and their blends absorbed very low amount of vegetable oil (Table 2). High viscosity of oil medium restricted its uptake into cross-linked network of polyurethanes. Much higher swelling of polyurethanes in small molecules solvents (chloroform and toluene) in compare to edible oils absorption was also noted by Zhang et al. (2017).

Environmental degradability of polymers is mainly affected by water (often containing degradative compounds) availability and by easy water molecules migration into the polymer structure. The presence of cross-links in the polymers network generally significantly reduces water penetration that is generally one of the reasons of lower degradability of cross-linked than linear polyurethanes.

**Fig. 1:**
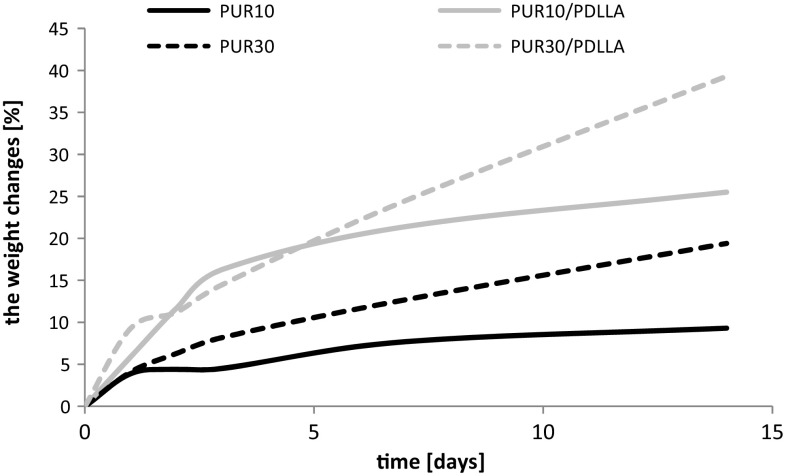
Water sorption by cross-linked polyurethanes and their blends with PDLLA. Data for PUR10 and PUR30 presented in (Brzeska et al.^2017^)

The synthetized polyurethanes and their blends were incubated in water for 14 days. In the case of samples with higher amount of R,S-PHB in soft segments (PUR30 and PUR30/PDLLA) the water sorption increased gradually in time and they did not achieve the visible level of saturation (Fig. 1). Using the higher amount of R,S-PHB for the polyurethane synthesis and blending with PDLLA significantly increased sorption of water by samples. Increasing hydrophilicity of linear polyesterurethanes after introducing R,S-PHB into their structure was described in previous papers (Brzeska et al. 2015c). Moreover for both blends: PUR10/PDLLA and PUR30/PDLLA adding a second component—PDLLA—resulted in higher water sorption in comparison to native PUR10 and PUR30.

**Fig. 2:**
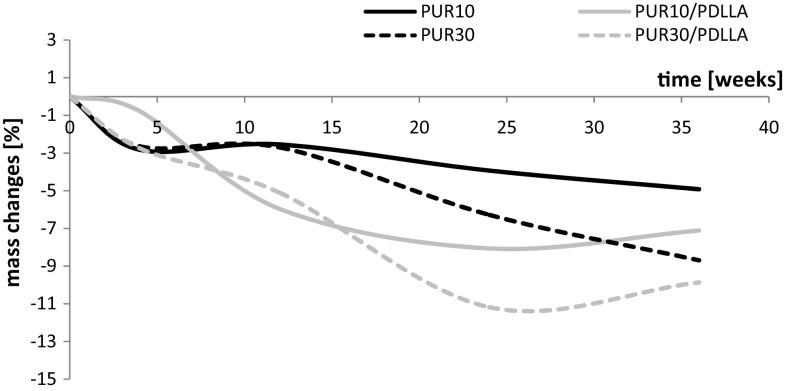
Weight changes of PURs and their blends with PDLLA after incubation in the buffer solution. Data for PUR10 and PUR30 presented in (Brzeska et al.^2017^)

**Fig. 3:**
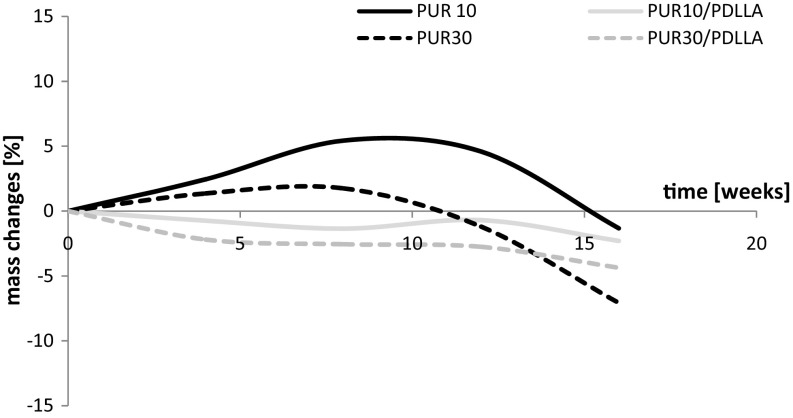
Weight changes of PURs and their blends with PDLLA after incubation in oxidative solutions. Data for PUR10 and PUR30 presented in (Brzeska et al. 2017)

The weight changes of samples of polyurethanes and their blends after incubation in the buffer solution are reported in Fig. 2, and after incubation in oxidative solution—in Fig. 3.

The rate of degradation of polyurethanes in hydrolytic conditions was slow. After 36 weeks of incubation in the buffer solution, the samples reduced their weight to 4.9 and 8.9%, respectively, for PUR 10 and PUR 30. As it was expected PUR 30 (with the higher amount of linear, almost amorphous R,S-PHB), with lower density and higher water sorption, degraded faster.

What is interesting the weight reduction of cross-linked PUR 10 was higher than linear polyurethane with similar construction of soft segments (but with PCL_diol_ instead of PCL_triol_) under hydrolytic conditions of phosphate buffer solution (Brzeska et al. 2015c). Decreased molecular mass of linear polyurethanes after 36 weeks incubation in buffer solutions indicated that macrochains were cut but not eluted from polymer bulk at this stage of degradation (Brzeska et al. 2015c).

As it was also expected, polyurethanes degradation under hydrolytic conditions was accelerated after blending with PDLLA. The changes were similar as in case of blends of linear polyurethanes (Brzeska et al. 2015c).

According to results of studies conducted by Christenson (Christenson et al. 2004) treatment with 20% hydrogen peroxide/0.1 M cobalt chloride solution at 37 °C reproduced the chemical and physical characteristics of in vivo degradation at an accelerated rate. So time of polyurethane samples incubation in oxidative solution was shorter (16 weeks) than in phosphate buffer solution (36 weeks). Despite that high reactivity of oxidative solution, the samples weight reduction at the end of investigation was small. So, it could be stated that polyurethanes and their composites with polylactide degraded faster in hydrolytic than in oxidative solution (Fig. 2). It indicated that the degradation process run mainly by hydrolysis of ester groups.

Polyurethanes and their blends with polylactide degraded in the oxidative solution slower than in hydrolytic conditions. It is well known that ester linkages are sensitive to hydrolytic attack, whereas other linkages are sensitive to oxidative agents. It was the reason why the investigated polyurethanes (with oligoesters in soft segments) and their blends (with ester linkages in the structure) degraded faster in the phosphate buffer than in the oxidative solution.

Introducing of PCL_triol_ into soft segments of polyurethanes moved its melting temperature (*T*_m1_) from 40.7 to about 54 °C and reduced the melting enthalpy (Δ*H*_1_) from 34.7 J/g to 9.1 and 15.9 J/g, respectively, for PUR 10 and PUR 30 (Table 3). The addition of more R,S-PHB into PUR 30 than into PUR 10 increased the mobility of PCL_triol_ chains and their ordering what consequently increased their crystallinity (despite the amorphous state of R,S-PHB).

**Table 3:** Thermal properties of polyurethanes and their blends with PDLLA before and after incubation in hydrolytic (PBS) and oxidative (OX) solutions

Sample	Incubation time (weeks)	*T*_m1_ (°C)	Δ*H*_1_ (J/g)	*T*_m2_ (°C)	Δ*H*_2_ (J/g)	*T*_m3_ (°C)	Δ*H*_3_ (J/g)
PUR 10	0*	54.2	9.1	88.1	1.8	121.4	0.60
PUR 10 (PBS)	12	57.6	7.5	97.4	5.9	–	–
	36*	60.6	12.3	99.0	5.3	–	–
PUR 10 (OX)	12	59.3	15.5	–	–	120.2	0.20
	16*	56.9	14.5	–	–	–	–
PUR 10/PDLLA	0	51.9	18.6	–	–	121.9	0.01
PUR 10/PDLLA (PBS)	12	61.5	15.4	–	–	120.1	0.20
	36	58.1	18.7	–	–	124.7	0.01
PUR 10/PDLLA (OX)	12	72.5	23.0	–	–	120.2	0.07
	16	58.5	12.6	–	–	125.0	0.03
PUR 30	0*	54.1	15.9	94.8	0.7	–	–
PUR 30 (PBS)	12	58.8	10.9	105.4	3.8	–	–
	36*	56.1	15.6	105.7	8.0	–	–
PUR 30 (OX)	12	61.3	12.9	–	–	119.9	0.30
	16*	57.7	11.2	–	–	120.6	0.20
PUR 30/PDLLA	0	54.7	19.2	–	–	125.2	0.02
PUR 30/PDLLA (PBS)	12	64.0	11.7	–	–	120.5	0.10
	36	54.1	18.9	–	–	124.4	0.10
PUR 30/PDLLA (OX)	12	61.7	9.0	–	–	120.2	0.06
	16	58.8	16.8	–	–	–	–

* Data presented in (Brzeska et al. 2017)

**Fig. 4:**
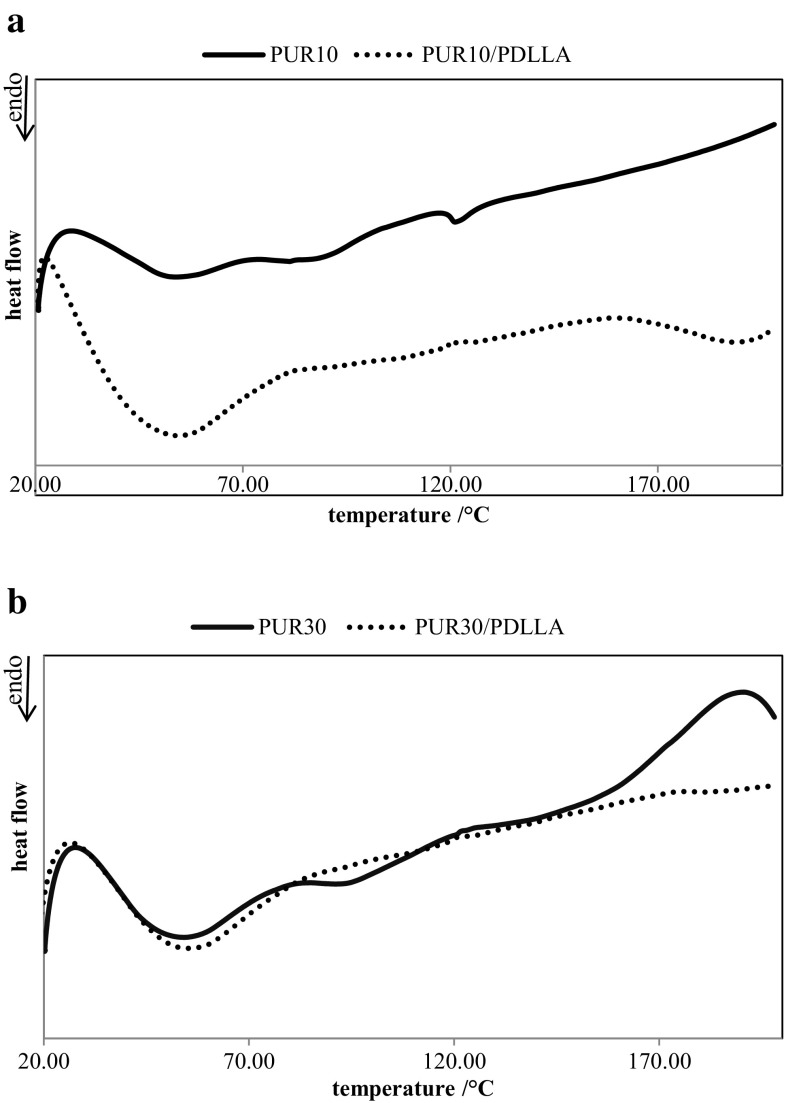
DSC thermograms of polyurethanes PUR 10 (**a**) and PUR 30 (**b**), and their blends with PDLLA

Blending the cross-linked polyurethane PUR 10 with PDLLA decreased the melting temperature of its soft segments. Chains of polylactide acted as plasticizers which caused reduction of the melting temperature (Fig. 4a). This was not observed for the sample with the higher amount of R,S-PHB (PUR 30), wherein blending the polyurethane with polylactide shifted slightly Tm_1_ to higher temperature on DSC thermograms (Fig. 4b).

In both cases, for PUR 10 and PUR 30, the presence of chains of amorphous polylactide visibly facilitated the ordering of polyurethane chains, which consequently caused an increase of soft segment crystallinity (increasing of Δ*H*_1_).

Increasing of R,S-PHB content in soft segments caused the greater distances between the cross-links network nodes (the amount of PCL_triol_ was reduced) and between the polyurethane chains (because of side methyl groups in R,S-PHB). This increased free space between the chains and plasticizing action of polylactide facilitated the water molecules penetration into the network of polyurethanes, despite the increase in crystallinity of PUR 30 and blends in comparison to PUR 10. It resulted in increasing of the amount of absorbed water by polyurethanes and their blends (Fig. 1).

After incubation of polyurethanes and their blends in both degradative environments, an increase in the melting temperature of soft segments was observed. It suggested that polyurethane macrochains were additionally cross-linked. Tendency of degraded polymers to further cross-link was especially visible during incubation in the oxidative solution, what caused the swelling of samples (Fig. 3).

Two processes were observed in soft segment structure after the incubation of samples in degradative solutions: (1) increasing of ΔH_1_ what suggested that amorphous phase degraded first (it caused that amount of crystalline phase increased) and (2) decreasing Δ*H*_1_ suggested that short polymer chains, created after degradation, were reorganized and cross-linked.

Table 4 shows AFM analysis of the microtopography of the polyurethanes and their blends with PDLLA before and after their incubation in oxidative and hydrolytic solutions. The average roughness (*R*_a_) of polymer surface was immersed in lower left corner of each picture.

**Table 4:**
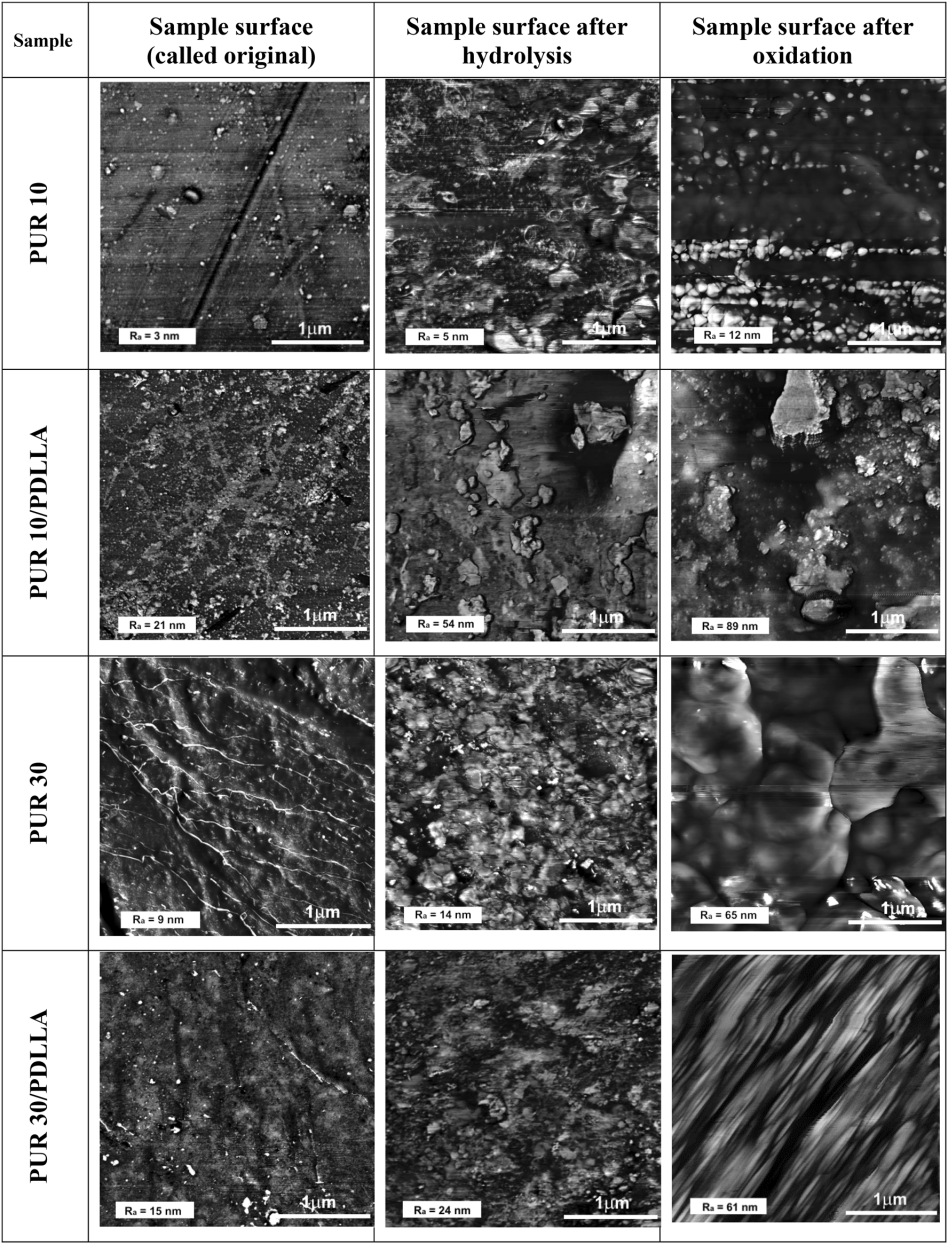
The surface of polyurethanes and their composites at AFM-phase image (5 × 5 μm for samples of PUR 30/PDLLA and 3 × 3 μm for other samples) before and after incubation in hydrolytic (36 weeks) and oxidative (16 weeks) solutions

Generally, the surface of native samples was quite smooth. The fact that the roughness of all samples surface was higher than for PUR 10 (*R*_a_ = 3 nm) could be associated with their higher crystallinity (Table 3). Increasing of R,S-PHB content in soft segments increased surface roughness from 3 to 9 nm, respectively, for PUR10 and PUR30. Also blending of polyurethanes with PDLLA had made their surface more rough.

AFM analysis confirmed that surfaces of PURs and their blends after incubation in hydrolytic and oxidative solutions became much rougher. It is known that degradation begins with the penetration of water into the polymer structure. In case of PDLLA and polyesterurethanes, it leads to hydrolysis of esters bonds primarily in the amorphous phase (Nowak and Pająk 2010). The short water-soluble polymer chains are formed and moved into the surrounding environment. When the rate of release of these oligomers is higher than the speed of water diffusion into the sample bulk, the surface erosion is visible (Nowak and Pająk 2010).

AFM-phase images showed that the surface of degraded samples was eroded (Table 4). The depth of depressions on the surface of the samples was much higher after their incubation in degradative solutions than before. Changes in surface roughness were significantly higher after incubation in aggressive oxidative than in hydrolytic solution.

Despite that the weight loss of degraded samples was not significant (Figs. 2, 3) the changes of thermal properties of polymers and changes of the surface topography indicated that cross-linked polyurethanes, based on synthetic poly([*R*,*S*]-3-hydroxybutyrate), and their blends with poly([d,l]-lactide) were susceptible to degradation.

## Conclusions

Cross-linked polyurethanes based on synthetic poly([*R*,*S*]-3-hydroxybutyrate) and their blends with poly([d,l]-lactide) appeared as degradable under hydrolytic and oxidative solutions. Because of very aggressive conditions of the oxidative solution, it was stated that polyurethanes degraded mainly through the hydrolysis. Increasing R,S-PHB amount in soft segments and blending polyurethanes with PDLLA introduced more ester linkages (sensitive to hydrolytic degradation) and increased the distance between the network nodes in cross-linked polyurethanes. It was the main reason of higher water sorption, and thus, higher degradability of polyurethane PUR 30 than PUR 10, and also blends with PDLLA than native PURs. Despite of small mass reduction of polyurethanes and their blends after incubation in degradative solutions, changes of thermal properties and surface erosion were observed.
